# Fish Oil Present in High-Fat Diet, Unlike Other Fats, Attenuates Oxidative Stress and Activates Autophagy in Murine Adipose Tissue

**DOI:** 10.3390/nu17233776

**Published:** 2025-12-01

**Authors:** Karolina Ciesielska, Jacek Wilczak, Adam Prostek, Piotr Karpiński, Rafał Sapierzyński, Alicja Majewska, Żaneta Dzięgelewska-Sokołowska, Małgorzata Gajewska

**Affiliations:** 1 Department of Physiological Sciences, Institute of Veterinary Medicine, Warsaw University of Life Sciences, 02-776 Warsaw, Poland; karolina_ciesielska@sggw.edu.pl (K.C.); jacek_wilczak@sggw.edu.pl (J.W.); adam_prostek@sggw.edu.pl (A.P.); alicja_majewska@sggw.edu.pl (A.M.); zaneta_dziegelewska-sokolowska@sggw.edu.pl (Ż.D.-S.); 2 Department of Technology and Food Safety, Faculty of Health Sciences, University of Lomza, 18-400 Łomża, Poland; pkarpinski@al.edu.pl; 3 Department of Pathology and Veterinary Diagnostics, Institute of Veterinary Medicine, Warsaw University of Life Sciences, 02-776 Warsaw, Poland; rafal_sapierzynski@sggw.edu.pl

**Keywords:** obesity, lard, coconut oil, olive oil, fish oil, visceral adipose tissue, subcutaneous adipose tissue, autophagic flux, total antioxidative status

## Abstract

**Background/Objectives:** Increased fat intake and high content of saturated fatty acids in the diet are associated with higher body weight and an increased risk of obesity. This study aimed to determine the impact of a high-fat diet (HFD) on white adipose tissue (WAT) metabolism and to verify whether this effect depends on the sources of lipids in HFD. **Methods:** Male C57BL/6J mice, 7 weeks old, received a control (Ctrl.) or high-fat diet (HFD) with 10% and 45% energy from fat, respectively, for 15 weeks. Lard was used as the main dietary fat in the HFD group. Next, the HFD group was subdivided into HFD-L, HFD-CO, HFD-OO and HFD-FO groups differing in the lipid sources (lard, coconut oil, olive oil, fish oil, respectively). The experiment was continued for 12 consecutive weeks. The study analyzed the concentration of different fatty acids in visceral (VAT) and subcutaneous (ScAT) adipose tissue; the levels of autophagy markers: beclin1, Atg5, LC3, p62, AMPK; ER stress markers: phos-PERK, CHOP, XBP-1 and oxidative stress parameters: TAS and TBARS in VAT and ScAT. **Results:** Mice in all HFD groups showed increased body mass and adipose tissue hypertrophy. Blood glucose concentration remained elevated in the HFD-L group but normalized in other HFD groups by the end of the dietary intervention. Fatty acid content in VAT and ScAT reflected the dietary sources in HFD. The HFD-L, HFD-CO, HFD-OO groups showed increased beclin1, ATG5, and p62 levels in VAT but the LC3-II/LC3-I ratio was similar to the control, suggesting impaired autophagic flux. In the HFD-FO group, the LC-II/LC-I ratio was elevated, along with decreased p62 levels, indicating active autophagic degradation. Changes in autophagy activity were insignificant in ScAT. ER stress markers were also mostly unaffected by HFD in both adipose tissue depots. TAS and TBARS values in VAT and ScAT were similar in the HFD-L and HFD-CO groups, and the HFD-OO group showed increased TAS and decreased TBARS, while the HFD-FO reduced TBARS. **Conclusions:** Antioxidant capacity and autophagy activity in WAT depended on fat content and lipid source, especially in the visceral depot. Fish oil induced changes in cellular metabolism, especially in VAT, diminishing the detrimental effects of HFD.

## 1. Introduction

Obesity and prolonged consumption of a high-fat diet (HFD) constitute critical factors contributing to the development of metabolic dysfunctions, including insulin resistance, type 2 diabetes and cognitive impairments [1,2,3]. Not only the quantity but also the type of dietary fat plays a pivotal role in shaping metabolic health. It is established that saturated and unsaturated fatty acids exert divergent effects. Diets rich in saturated fatty acids (SFAs) are more likely to trigger oxidative stress, inflammation and endoplasmic reticulum (ER) stress [4,5], whereas diets with higher contents of monounsaturated (MUFA) or polyunsaturated fatty acids (PUFAs) show anti-apoptotic effect and inhibition of ER stress [6].

Current approaches for weight reduction, which encompass physical activity, pharmacological interventions, surgical procedures, and various lifestyle strategies, often pose challenges in terms of adherence, and may be accompanied by significant side effects [7]. Recently, attention has shifted toward targeting metabolically active tissues, particularly white adipose tissue (WAT), which serves both as a lipid reservoir and a dynamic endocrine–metabolic organ [8]. Two major WAT depots, visceral adipose tissue (VAT) and subcutaneous adipose tissue (ScAT), differ in their contributions to metabolic dysfunctions. VAT, compared with ScAT, is more cellular, vascularized, immune-cell rich, and metabolically active; increased size of the visceral depot thereby contributes more strongly to insulin resistance, inflammation and obesity-related diseases, such as type 2 diabetes [9]. Excessive lipid intake, particularly SFA, promotes adipocyte hypertrophy, impaired lipid storage, mitochondrial dysfunction, leading to reactive oxygen species (ROS) generation and unfolded protein response (UPR) activation, resulting in ER stress. These processes trigger inflammation, oxidative stress and apoptotic pathways, which link hypertrophic adipocytes to metabolic dysfunction. In obesity, adipose tissue exhibits increased secretion of pro-inflammatory cytokines (tumor necrosis factor alpha (TNF-α), interleukins: IL-6, IL-8) and reduced release of anti-inflammatory mediators (IL-10), which lead to chronic low-grade inflammation [9]. Several studies have also demonstrated increased autophagic activity in the adipose tissue of obese individuals, evidenced by a high number of autophagosomes and autolysosomes within adipocyte cytoplasm [10,11]. UPR activation, ER and oxidative stress may modulate autophagy activity in cells, but the exact role of enhanced autophagy in hypertrophic adipose tissue has not been unambiguously explained so far [9].

Dietary fatty acids have emerged as potential modulators of adipocyte metabolism, capable of influencing lipid storage and the endocrine functions of adipocytes. Several studies have addressed how different sources of dietary fats modulate autophagy, oxidative stress and ER stress in different tissues and organs of obese individuals. In vitro studies showed that long-chain saturated fatty acids (LC-SFA), such as palmitic acid (PA) and stearic acid (SA) induced ER stress by increasing the expression of C/EBP homologous protein (CHOP) in the liver cells [4]. In another study, palmitic acid induced ER stress, leading to the activation of the c-Jun *N*-terminal kinase (JNK) signaling pathway, and causing autophagy induction in 3T3-L1 murine adipocytes [12]. Autophagy activation via JNK signaling pathway decreased PA-induced inflammation, suggesting that enhancing autophagy could alleviate adipocyte dysfunction and inflammatory response [12]. Moreover, in 3T3-L1 adipocytes PA not only promoted inflammation but also caused reduced glucose utilization [13]. These results highlight the detrimental effect of LC-SFA on adipocyte metabolism.

Unlike LC-SFA, medium-chain fatty acids (MC-SFA) present in coconut oil (CO), have shown variable and less predictable effects. In rats, supplementation with CO or virgin coconut oil (VCO) during high-fat diet feeding yielded contrasting effects [14]. Partial replacement with CO reduced weight gain and improved hepatic antioxidant status despite persistent liver steatosis and triglyceride accumulation, indicating enhanced antioxidant defense without preventing lipid deposition [15]. In turn, VCO supplementation increased weight gain, LDL cholesterol level, aspartate aminotransferase (AST) and alanine aminotransferase (ALT) activity in blood. In addition, HFD enriched with VCO resulted in hepatic lipid accumulation and adipocyte hypertrophy with elevated TNF-α expression, suggesting exacerbation of HFD-induced metabolic alterations, adipose inflammation, and liver lipid deposition [14].

Studies have shown that some lipids present in olive oil (OO), such as oleanolic acid, which structurally belongs to pentacyclic triterpenoids, regulate body weight, glycemia, and insulin sensitivity primarily through the modulation of glucose production and uptake, insulin signaling in liver and adipose tissue, and activation of peroxisome proliferator-activated receptor (PPARγ/α). Antioxidant and anti-inflammatory effects of oleanolic acid, including inhibition of pro-inflammatory cytokines secretion, NLRP3 inflammasome signaling, and promotion of M2 macrophage polarization, contribute to its anti-obesity actions [16]. It is also well-documented that oleic acid, one of the main MUFA found in olive oil, prevents palmitate-induced ER stress, NF-κB-mediated inflammation, and insulin resistance by maintaining AMP-dependent protein kinase (AMPK) activation in skeletal muscle cells [17]. Furthermore, partial replacement of a high-fat diet with olive oil reverses steatosis, ER stress, apoptosis, and autophagy impairment in nonalcoholic fatty liver disease models, underscoring the ER stress–autophagy axis’s vital role in oleic acid-mediated liver protection [18].

PUFAs are increasingly investigated for their potent anti-inflammatory effects, which are advantageous in mitigating obesity-related metabolic dysfunction. PUFAs from various fish oils have been shown to improve glucose and lipid metabolism and reduce systemic inflammation, suggesting a potential therapeutic role in metabolic disorders [19]. Long-chain *n*-3 PUFAs, particularly docosahexaenoic acid (DHA) and eicosapentaenoic acid (EPA), can enhance the secretion of adiponectin, a key anti-inflammatory and insulin-sensitizing adipokine, from adipocytes. In 3T3-L1 adipocytes, DHA increased both adiponectin mRNA expression and protein concentration via a PPARγ-dependent mechanism, while EPA primarily stimulated adiponectin secretion, highlighting distinct actions of these fatty acids on adipocyte function [20]. DHA and EPA also attenuated ER stress induced in human coronary artery endothelial cells. In cells with induced ER stress, DHA decreased UPR markers, such as inositol-requiring 1 alpha (IRE1α) and protein kinase RNA-like ER kinase (PERK), while enhancing glucose-regulated protein 78 (GRP78) expression [21]. Beyond these effects, fish oil-derived omega-3 PUFAs, particularly EPA and DHA, activate AMPK and increase Peroxisome Proliferator-Activated Receptor Gamma Coactivator 1 Alpha (PGC-1α) protein levels while attenuating muscle catabolism, restoring the balance between protein synthesis and degradation in obese rats [22]. Engagement of AMPK/PGC-1α signaling has been demonstrated to stimulate autophagic flux, improve mitochondrial quality control, and mitigate oxidative stress, thereby maintaining skeletal muscle mass and regulating lipid metabolism.

Despite the growing body of evidence showing distinct effects of different lipids on the physiology and metabolism of various tissues and organs, research specifically addressing adipose tissue responses to these lipids is still insufficient. Even less is known about the effect of different dietary lipids on hypertrophic adipose tissue, when the hypertrophy had been previously induced by the consumption of high-fat diet rich in saturated fatty acids. Therefore, in the present study, we hypothesized that the hypertrophic adipose tissue may show differences in metabolism when the source of lipids in HFD are replaced, providing diverse types of fatty acids: MC-SFA, MUFA or PUFA. To verify the hypothesis, we used a murine model of diet-induced obesity (DIO) in which C57BL/6J mice were first fed an HFD containing lard as the main source of exogenous, dietary fatty acids for 15 weeks to induce adipose tissue hypertrophy, following a 12 week period of dietary intervention, during which the animals received HFD, but differing in the main source of lipids: lard, coconut oil, olive oil or fish oil. After 27 weeks of the in vivo experiment, we analyzed the concentration of different fatty acids in VAT and ScAT, autophagy activity in both types of analyzed white adipose tissue, the level of chosen ER stress markers (phos-PERK, CHOP, XBP-1) and oxidative stress in VAT and ScAT. This approach aimed to delineate how dietary fatty acid composition modulates depot-specific metabolic stress in hypertrophic adipose tissue.

## 2. Materials and Methods

### 2.1. Animals and Diet Intervention

**Figure 1:**
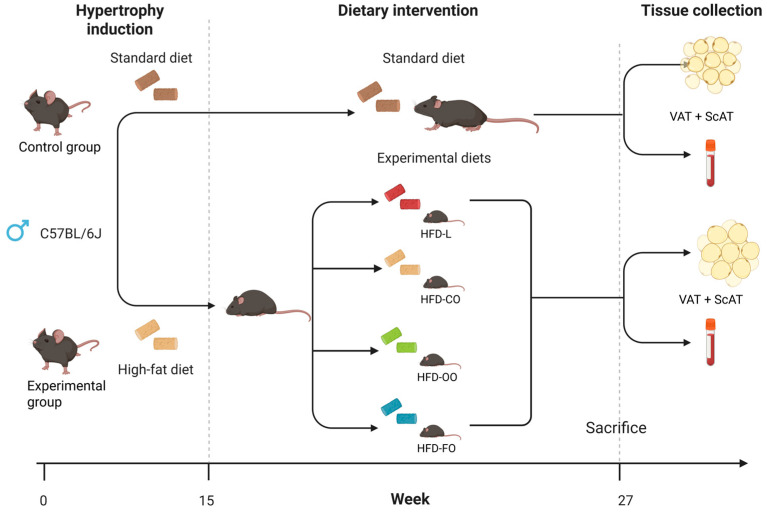
Scheme of in vivo experiment on mice. The study comprised two sequential phases. Phase 1—Diet-Induced Obesity (15 weeks): Fifty-four male C57BL/6J mice were fed a high-fat diet (HFD) providing 45% of energy (kcal) from fat to induce obesity-related phenotype. An additional control group (*n* = 18) received a standard purified feed for mice. Body weight and glucose levels were monitored weekly. After 15 weeks, 6 animals per group were euthanized for blood and tissue collection. Phase 2—Exposure to distinct dietary lipid sources (12 weeks): The remaining mice were randomly assigned to four experimental HFD groups (*n* = 12 each), differing in lipid source—lard (HFD-L), coconut oil (HFD-CO), olive oil (HFD-OO), and fish oil (HFD-FO). The control group (*n* = 12) continued receiving the standard feed. Body weight, glucose levels, and food intake were monitored throughout the dietary intervention.All diets (control and HFDs) were produced as pellets by ZooLab (Sędziszów, Poland), following the nutritional recommendation for rodents. The fat used in the production of specific diets was provided by the following producers: AGII Sp.z o.o., Pępowo, Poland—lard; BASSO FEDELE and FIGLI, S. Michele de Serano, Italy—olive oil and soybean oil; CD S.A., Bielsko-Biała, Poland—coconut oil; HOLISTA Sp.z o.o., Myślenice, Poland—cod liver oil. The composition of all diets is shown in detail in Table 1. The composition of fatty acids in all diets is presented in Table 2. The second phase of the dietary intervention was carried out for 12 consecutive weeks. The weight and non-fasting blood glucose concentration in the mice was recorded once every week. To ensure that observations took place in a state of chronic obesity, animals that did not show the expected weight gain (~15 g above what a matched control would gain over the experimental period) were excluded from the experiment. Based on this criterion, two mice were excluded before the end of the second phase of the in vivo experiment: one from the HFD-CO group and one from the HFD-FO group. No adverse effects were observed in mice during both phases of the dietary intervention.

The experiment was performed on 72 male C57BL/6j mice (6 weeks old) purchased from Charles River Laboratories (Sulzfeld, Germany). All procedures were approved by the 2nd Local Ethics Committee in Warsaw (Resolution No. WAW2/081/2022; date of approval: 6 July 2022) in accordance with Polish law and the EU Directive 2010/63/EU for animal experiments. The experiment was conducted at the animal house of the Department of Immunology Medical University of Warsaw. Animals were housed in groups of 6 mice per cage in a temperature-controlled room under a 12 h light–dark cycle with ad libitum access to water and food. After one week of acclimatization to the animal house conditions, mice were randomly separated into two main groups: experimental group (*n* = 54)—mice fed a high-fat diet containing 45% of energy (kcal) from fat (40% kcal from lard, 5% kcal from soybean oil) and control group (*n* = 18)—mice fed a standard purified feed for mice, comprising 10% of energy from fat (6% kcal from soybean oil, 4% kcal from lard). The first phase of the dietary intervention, aiming to induce overweight/obesity in mice in the experimental group, was carried out for 15 weeks (Figure 1). After 15 weeks, 6 animals from both groups were sacrificed and blood and tissues were collected for further analyses (body mass, blood glucose levels, fatty acids profile in VAT, ScAT and blood plasma). The remaining animals from the experimental group were divided into 4 HFD groups (12 animals each). In the second phase of the experiment, these mice were fed HFD comprising different sources of fat, as follows: HFD-L group—high-fat diet containing 40% kcal from lard, 5% kcal from soybean oil; HFD-CO—high-fat diet containing 30% kcal from coconut oil, 10% kcal from lard, 5% kcal from soybean oil, HFD-OO—high-fat diet containing 30% kcal from olive oil, 10% kcal from lard, 5% kcal from soybean oil, HFD-FO—high-fat diet containing 30% kcal from fish oil (cod liver oil), 10% kcal from lard, 5% kcal from soybean oil. The control group (Ctrl., *n* = 12) continued to receive the control standard purified feed (Figure 1). Sample-size determination was based on the primary endpoint of leptin concentration in blood. Power analysis was conducted a priori assuming a baseline mean of 15%, an expected increase to 25%, variance of 19%, power = 0.80, and α = 0.05 (two-tailed). The required sample size was calculated using the formula: *n* ≥ [(tγ + t_1_ − α/2)^2^ × s^2^]/(μ_1_ − μ_0_)^2^, with tγ = 0.68 and t_1_ − α/2 = 1.96. This yielded *n* = 12.43; therefore, 12 animals per group were selected in the main part of the in vivo experiment (the second phase) to meet the minimum requirement. This sample size provided ≥80% power to detect the expected effect size while ensuring sufficient biological material for planned metabolomic, biochemical, and colorimetric analyses. The sample-size calculations were performed according to standard power-analysis methodology for normally distributed outcomes.

After being fed different HFDs or the control diet for 12 weeks, the mice were anesthetized through intraperitoneal injection with a solution of ketamine (87 mg/kg of body mass) and xylazine (7.5 mg/kg of body mass), and an intracardiac puncture was immediately performed to collect fresh blood samples. The mice were sacrificed by cervical dislocation. The abdominal visceral white adipose tissue (VAT), and subcutaneous adipose tissue (ScAT) from the inguinal subcutaneous fat depot located near the hind limbs were collected by snap freezing in liquid nitrogen or by fixing the tissue samples in 10% buffered formaldehyde, followed by embedding the tissue samples in paraffin for histological evaluation.

In the present study, randomization was provided by random assignment of animals to experimental groups (Control, HFD-L, HFD-CO, HFD-OO, HFD-FO) using computer-generated random numbers, ensuring equal group sizes (6 animals per cage). Allocation was performed after baseline body weight measurement, and animals were distributed so that mean body weight did not differ systematically between groups at the start of the first and the second phase of the experiment. Each cage contained animals from a single treatment group. The staff of the Department of Immunology Medical University of Warsaw, who were responsible for the daily care of animals subjected to the in vivo experiment, were aware of the mouse allocation to ensure provision of the correct diet.

### 2.2. Fatty Acid Composition

The fatty acid (FA) composition of feeds and adipose tissue samples was determined by gas chromatography coupled with mass spectrometry (GC–MS), following standardized procedures [Polish national standard: PN-ISO 5509:2001—Animal and vegetable fats and oils—Preparation of methyl esters of fatty acids; PN-EN ISO 5508:1996—Animal and vegetable fats and oils—Analysis by gas chromatography of methyl esters of fatty acids, Publisher: Polish Committee for Standardization] and the method described by Hara and Radin [23]. Tissue samples from six animals per group were analyzed by GC-MS.

Total lipids were extracted from feed and tissue samples using a hexane–isopropanol mixture (3:2, *v*/*v*), as described by Hara and Radin (1978) [23]. Fatty acid methyl esters (FAMEs) were then prepared according to PN-ISO 5509:2001.

The separation and identification of FAME were performed using a GCMS-QP2020 NX system (Shimadzu, Kyoto, Japan) equipped with an AOC-6000 RTC autosampler and an SH-2330 capillary column (30 m × 0.25 mm i.d., 0.25 μm film thickness). The injector temperature was set at 250 °C. The oven temperature program was as follows: initial temperature 40 °C (1 min), ramped at 15 °C/min to 150 °C, then at 8 °C/min to 240 °C and held for 3 min. Helium was used as the carrier gas at a flow rate of 1.60 mL/min with a split ratio of 1:10. The transfer line and ion source temperatures were both set at 250 °C. The mass spectrometer operated in total ion current (TIC) mode, scanning a mass range of 50–500 Da.

Fatty acids were identified by comparing their retention times and mass spectra with reference standards (NCP-GLC-674, NCP-GLC-37, NCP-GLC-96, NCP-GLC-490; Nu-Chek Prep Inc., Elysian, MN, USA). The FA content was expressed as mg of fatty acid per g of feed and mg of fatty acid per 100 mg of adipose tissue.

### 2.3. Histopathological Assessment of Visceral and Subcutaneous Adipose Tissue

Samples of visceral, as well as subcutaneous, adipose tissues from each mouse were fixed using 10% neutral buffered formalin, processed through standard protocols (dehydration, clearing and paraffinization). The sections from each paraffin blocks were cut into 4 µm-thick slides and stained with hematoxylin–eosin. The microscopic examination was performed by a veterinarian pathologist using the light microscope Olympus BX43 (Olympus, Tokyo, Japan). Light microscope images obtained from the light microscope were additionally analyzed by ImageJ.JS v0.6.0 software (National Institutes of Health and the Laboratory for Optical and Computational Instrumentation, University of Wisconsin). The size of single adipocytes in each slide from VAT and ScAT tissue sections was determined by calculating an area of each adipocyte and expressed in µm^2^ units. At least 50 adipocytes per slide from control and each experimental group were measured.

### 2.4. Immunoblotting Analyses

Protein extraction from visceral and subcutaneous adipose tissue was performed by homogenization of 300 mg of tissue samples in Pierce^TM^ RIPA buffer (cat. no. 89900, Thermo Fisher Scientific, Waltham, MA, USA) supplemented with protease inhibitor cocktail (cat. no. P8340) and phosphatase inhibitor cocktail (cat. no. P5726) (Merck/Sigma-Aldrich, Darmstadt, Germany), followed by sample incubation on ice for 30 min. Next, samples were centrifuged at 20,000× *g* for 20 min, and supernatants containing extracted proteins were collected. Total protein concentration in samples was determined using Bio-Rad Protein Assay Dye Reagent according to the producer’s protocol (Bio-Rad Laboratories Inc., Hercules, CA, USA). Proteins (50 μg) were resolved by SDS-PAGE and transferred onto low-fluorescence PVDF membranes (Merck/Sigma-Aldrich, Darmstadt, Germany). For immunostaining, membranes were blocked with 5% nonfat dry milk in TBS buffer (20 mM Tris-HCL, 500 mM NaCl) enriched with 0.5% Tween20, thus called a TBST buffer. The membranes were incubated at 4 °C overnight with primary antibodies diluted in TBST buffer with 5% bovine serum albumin (cat. no. P7030; Merck/Sigma-Aldrich, Darmstadt, Germany). GAPDH was used as a reference protein. The next day, membranes were washed three times in TBST buffer and incubated with appropriate secondary antibodies conjugated with IR fluorophores: IRDye^®^ 680 or IRDye^®^ 800 CW, diluted with TBST buffer. Detailed information about all antibodies used in this study are presented in Table 3. The antibodies were purchased from the following producers: Cell Signaling Technology (Danvers, MA, USA), Invitrogen, Thermo Fisher Scientific (Waltham, MA, USA), **LI-COR (Lincoln, NE, USA).** ChemiDoc^TM^ MP Imaging System (Bio-Rad Laboratories, Hercules, CA, USA) was used to analyze the protein expression. Densitometric analysis was performed using Image Lab 6.1 software (Bio-Rad, Hercules, CA, USA). Immunoblot analysis was performed in six replicates (samples collected from six animals per group).

### 2.5. Analysis of Oxidative Stress Markers

Visceral and subcutaneous adipose tissue samples from at least 10 animals per group were homogenized in PBS, vortexed for 15 min, and centrifuged at 500× *g* for 15 min. The supernatants were used for analyses. Total antioxidant potential (TAS) was measured using the commercial Randox reagent kit (catalog number NX 2332). Thiobarbituric acid-reactive substances (TBARSs) were determined in both types of the adipose tissue according to the method described in detail by Aguilar Diaz De Leon and Chad R Borges [24].

### 2.6. Statistical Analysis

Statistical analysis was performed using GraphPad PrismTM version 7.00 software (GraphPad Software, Inc., La Jolla, CA, USA). Normality of data was analyzed with the Shapiro–Wilk test. One-way analysis of variance (ANOVA) with Tukey’s multiple comparison post-test was used to determine the significance of effects between the experimental groups. In the case of analyzing the adipocyte size, the data obtained did not show normal distribution in the Shapiro–Wilk test. Thus, a nonparametric Kruskal–Wallis test with Dunn’s multiple comparison post-test was used to determine statistical differences among tested groups. *p* < 0.05 was considered statistically different. Values in each graph are expressed as mean ± standard deviation (SD). Symbols on the graphs indicate significant differences between the specified groups, as follows: * *p* < 0.05; ** *p* < 0.01; *** *p* < 0.001, **** *p* < 0.0001.

## 3. Results

### 3.1. Changes in Body Mass and Glucose Levels in Mice

**Figure 2:**
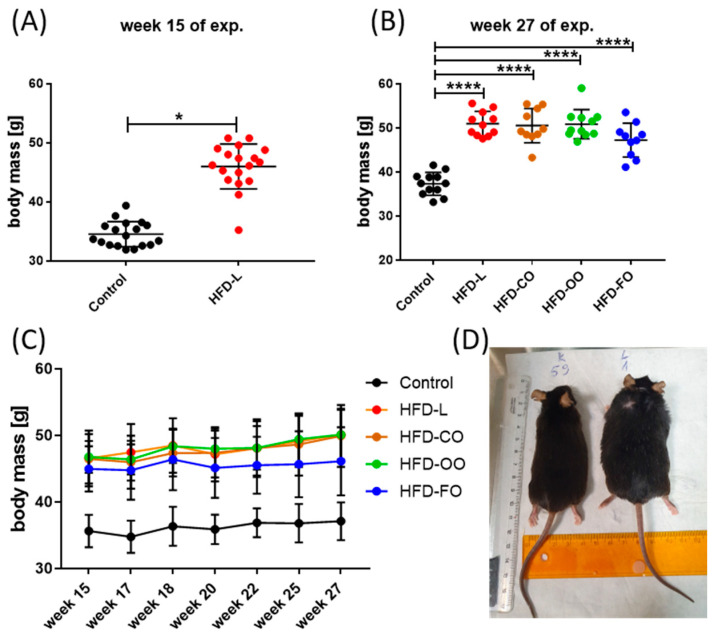
Changes in the body mass of mice during the DIO experiment; (**A**) body mass changes by the end of the first phase of the experiment, in which obesity was induced by HFD-L; (**B**) body mass by the end of the second phase of the experiment, in which animals in experimental groups received HFD containing different fat sources; (**C**) changes in the body mass of mice during the second experimental phase. Results are presented as means ± SD. Symbols * and **** show significant differences (* *p* < 0.05 or **** *p* < 0.0001) between the specified groups. (**D**) An image of mice from control and (K59) and HFD-L groups (L1) illustrating the differences in their body size by the end of the experiment.

The applied experimental model effectively induced obesity in mice. During the initial 15-week DIO phase, mice that were fed the HFD containing 45% kcal from fat, of which 40% kcal came from lard, showed a significantly higher body mass (*p* < 0.0001) compared with the mice receiving the standard, control diet that contained 10% of energy from fat (Figure 2A). During the subsequent 12-week dietary intervention phase, in which animals in experimental groups received HFD composed of different lipid sources, the body mass of mice in all experimental groups increased progressively and was constantly significantly higher compared with the body mass of animals in the control group (*p* < 0.0001) (Figure 2B–D). Mice that were fed the high-fat diet enriched with lard (HFD-L), coconut oil (HFD-CO) or olive oil (HFD-OO) displayed a similar increase in the body mass observed with time, whereas animals receiving the fish oil-based diet (HFD-FO) showed a tendency toward a lower weight gain; however, the difference in the body mass of mice in HFD-FO group and other HFD groups was not significant (HFD-L vs. HFD-FO: *p* = 0.09; HFD-OO vs. HFD-FO: *p* = 0.17; HFD-CO vs. HFD-FO: *p* = 0.10) (Figure 2C). No marked differences in feed consumption were noted among the animals from different experimental HFD groups and the control group (an average feed consumption of 2.8 g ± 0.2g/per mice/day was registered). Collectively, the data demonstrate the obesogenic effect of the high-fat diet and the potential modulatory role of dietary lipid composition on long-term weight gain.

**Figure 3:**
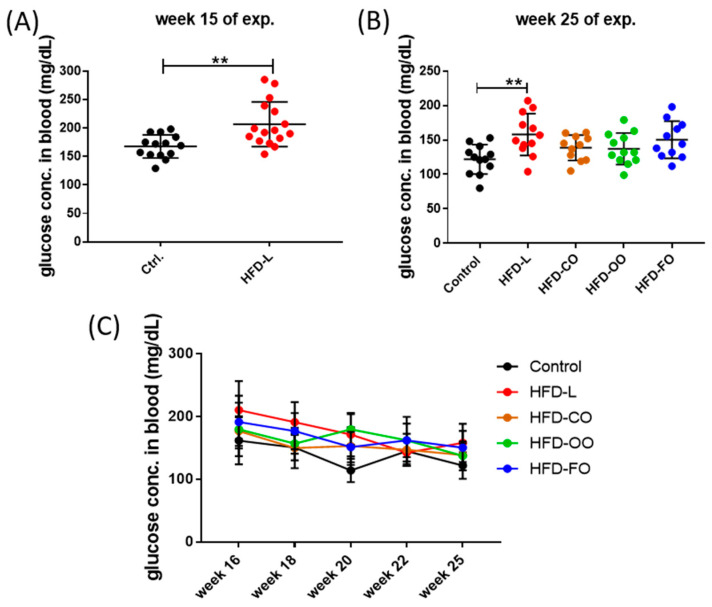
Non-fasting blood glucose levels in mice by the end of the first (**A**) and second (**B**) phase of the in vivo experiment; (**C**) changes in the non-fasting blood glucose concentration in the course of the second phases of the experiment. Results are presented as means ± SD. The symbol ** shows significant differences, *p* < 0.01 between the specified groups.

HFD also influenced the blood glucose levels in mice. In the first phase of the experiment (Figure 3A), non-fasting glucose levels were significantly higher in animals from the HFD group compared to control mice (*p* = 0.02). In the second phase of the experiment, distinct patterns of blood glucose levels emerged depending on the lipid source (Figure 3B,C). The HFD-L group showed sustained, significantly elevated non-fasting blood glucose concentrations in comparison to control group (*p* = 0.006). The mean concentration of glucose in mice from the HFD-L group was 158.0 ± 30.3 mg/dL by the end of the dietary intervention, whereas in the control group, the glucose concentration was 121.9 ± 21.3 mg/dL (Figure 3C), suggesting a prediabetic state in animals from the HFD-L group. However, a glucose tolerance test or measurement of insulin levels were not performed during this experiment, so the diabetogenic effect of the diet enriched with lard (HFD-L) could not be unambiguously confirmed in this study. The mean glucose concentration in the HFD-FO group was also above 150 mg/dL (150.3 ± 27.0 mg/dL), but this result did not reach statistical significance when compared to the control group (*p* = 0.055). Interestingly, the HFD-CO and HFD-OO groups presented a tendency to have moderately reduced glucose concentration values by the end of the experiment (138.7 ± 18.5 and 137.3 ± 22.9, respectively) compared with HFD-L, though still higher than in the control group (Figure 3B,C).

### 3.2. Histopathology Assessment of Visceral and Subcutaneous Adipose Tissue

**Figure 4:**
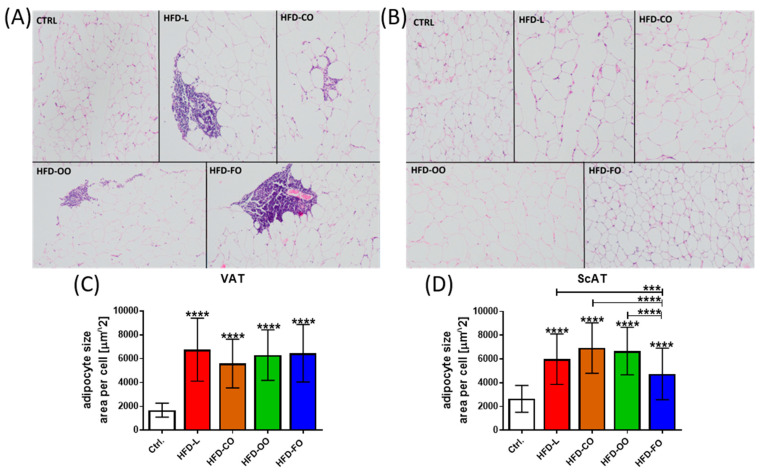
(**A**) Histopathological view of representative samples of visceral adipose tissue (VAT) of mice in specific experimental groups: CTRL—no pathological lesions; HFD-L—focal accumulation of small lymphocytes in visceral adipose tissue is visible; HFD-CO—focal infiltration of visceral adipose tissue by small lymphocytes; HFD-OO—focal accumulation of small lymphocytes in visceral adipose tissue is visible; HFD-FO—focal perivascular accumulation of small lymphocytes in visceral adipose tissue is visible. (**B**) Histopathological view of representative samples of subcutaneous adipose tissue (ScAT) of mice from specific experimental groups (CTRL, HFD-L, HFD-CO, HFD-OO, HFD-FO)—none of the animals in all the examined groups had pathological changes in the subcutaneous adipose tissue. Both panels (**A**,**B**) present hematoxylin–eosin staining. Images were taken at a magnification of 200×. (**C**,**D**) Size of adipocytes in VAT and ScAT measured by ImageJ and expressed as area [µm^2^] of single adipocytes in each analyzed slide. At least 50 adipocytes per slide from the control and each experimental group were measured. Results are presented as means ± SD. In graph (**C**), the symbol **** shows a significant difference *p* < 0.0001 between the specified HFD group and the control group. In graph (**D**), the symbol ****directly above the bars shows a significant difference *p* < 0.0001 between the specified HFD group and the control group; the symbols *** or **** above a line placed above the bars show a significant difference (*p* < 0.001 or *p* < 0.0001, respectively) between specific experimental HFD groups.

No pathological changes were observed in visceral adipose tissue samples taken from mice from the control group. Inflammatory changes (focal lymphocytic inflammation) were observed in the VAT of mice in the HFD groups. Such changes were observed in one out of five individuals in the HFD-L and HFD-CO groups, in two out of five mice in the HFD-OO group, and in three out of six individuals in the HFD-FO group (Figure 4A). In addition, the size of single adipocytes in each tissue sample from VAT was determined by calculating the area of each adipocyte by ImageJ software. The results revealed a significant difference between the samples from the experimental groups and the control group (*p* < 0.0001) (Figure 4C). Adipocytes from all HFD groups were significantly larger than the fat cells from control VAT, with no significant differences observed among the HFD groups. This proves that adipocytes in the VAT of animals that were fed the high-fat diet were hypertrophic. With subcutaneous adipose tissue, no pathological findings were observed in the analyzed tissue samples from the control and experimental HFD groups of mice (Figure 4B). However, adipocytes in ScAT of mice that were fed the HFDs were also significantly larger than those in the subcutaneous adipose tissue of control animals (*p* < 0.0001) (Figure 4D). Interestingly, the adipocytes in ScAT of mice receiving the HFD-FO diet were significantly smaller than the fat cells in other experimental groups (HFD-L vs. HFD-FO, *p* < 0.0008; HFD-CO vs. HFD-FO, *p* < 0.0001; HFD-OO vs. HFD-FO, *p* < 0.0001), suggesting that the fish oil content in the high-fat diet reduced the hypertrophy of ScAT in mice within the 12 weeks of the dietary intervention.

### 3.3. Fatty Acid Composition in Visceral and Subcutaneous Adipose Tissue

Histopathologic examination of VAT and ScAT confirmed hypertrophy of adipocytes in mice that were fed the HFD composed of different dietary lipids. Thus, we also analyzed the composition of fatty acids in both types of adipose tissue in samples from experimental groups and the control group using gas chromatography coupled with mass spectrometry. The results obtained are presented in Table 4 and Table 5. The composition of fatty acids (FAs) in VAT and ScAT reflected the FA composition found in the experimental and control feed given to mice (presented in Table 2). The control diet presented a balanced fatty acid composition, with intermediate levels of LC-SFA, MUFA, and PUFA. It contained palmitic acid (C16:0; 46.46 mg/g), stearic acid (C18:0; 59.03 mg/g), oleic acid (C18:1n9; 159.66 mg/g), linoleic acid (C18:2n6; 83.43 mg/g), and α-linolenic acid (ALA) (C18:3n3; 9.18 mg/g), but lacked detectable levels of long-chain omega-3 fatty acids. The HFD-L diet based on lard that was used in both phases of the dietary intervention was primarily composed of LC-SFA and MUFA, with high concentrations of palmitic acid (C16:0; 90.13 mg/g), stearic acid (C18:0; 67.39 mg/g), and oleic acid (C18:1n9; 188.48 mg/g). This diet contained relatively low levels of PUFA, including linoleic acid (C18:2n6; 49.27 mg/g) and ALA (C18:3n3; 4.87 mg/g), and was devoid of long-chain *n*-3 PUFA. The HFD-CO diet, containing coconut oil as the primary lipid source, was characterized by a high content of MC-SFAs, particularly lauric acid (C12:0; 105.78 mg/g), caprylic acid (C8:0; 14.26 mg/g) and capric acid (C10:0; 12.77 mg/g). This diet also contained myristic acid (C14:0; 48.99 mg/g), which was not detected in any other HFD used in the experiment. MUFA and PUFA levels were relatively low, and long-chain *n*-3 fatty acids were absent. The HFD-OO diet, enriched with olive oil, contained the highest concentration of oleic acid (C18:1n9; 212.56 mg/g). In addition, it included moderate levels of palmitic acid (C16:0; 31.25 mg/g), stearic acid (C18:0; 33.45 mg/g), and essential PUFAs such as linoleic acid (C18:2n6; 56.58 mg/g) and ALA (C18:3n3; 6.71 mg/g). No long-chain *n*-3 fatty acids were detected in this diet. In contrast, the HFD-FO diet, based on cod liver oil, exhibited a markedly different lipid profile, characterized by a high content of long-chain *n*-3 PUFA. Notably, it contained docosahexaenoic acid (DHA, C22:6n3; 48.67 mg/g), eicosapentaenoic acid (EPA, C20:5; 21.16 mg/g), and docosapentaenoic acid (DPA, C22:5n3; 7.31 mg/g). It also included elevated levels of palmitoleic acid (C16:1n7; 24.19 mg/g), arachidonic acid (C20:4; 40.16 mg/g), and moderate amounts of other MUFA and PUFA. These distinct fatty acid compositions in the murine feed encouraged us to analyze the content of FA in the adipose tissue of the mice taking part in the experiment.

The composition of FA in VAT and ScAT from animals in different experimental groups reflected the composition of specific diets, although some differences could be noted between the two types of adipose tissue. VAT in control mice showed the highest concentration of palmitic acid (C16:0; 7.521 ± 0.165 mg/100 mg tissue), palmitoleic acid (C16:7; 7.26 ± 1.123 mg/100 mg tissue), linoleic acid (C18:2n6; 14.666 ± 1.627 mg/100 mg tissue) and ALA (C18:3n3; 0.687 ± 0.083 mg/100 mg tissue) in comparison to VAT samples from all experimental groups. In control ScAT samples, only palmitoleic acid (C16:7; 6.186 ± 0.968 mg/100 mg tissue) and ALA (C18:3n3; 0.687 ± 0.083 mg/100 mg tissue) exhibited higher concentrations than in the HFD groups. Adipose tissue in mice that were fed the HFD-L diet contained significantly higher amounts of stearic acid (C18:0; VAT: 2.438 ± 0.235; ScAT: 3.010 ± 0.500 mg/100 mg tissue) than in all other experimental groups and the control group. In parallel, animals in the HFD-L group showed significantly lower content of ALA in both analyzed adipose tissue types compared to control (C18:3n3; VAT: 0.338 ± 0.082; ScAT: 0.380 ± 0.123 mg/100 mg tissue), whereas the concentration of linoleic acid was significantly lower than in the control in VAT (C18:2n6; 12.146 ± 0.507 mg/100 mg tissue), but significantly higher than in the control in ScAT (13.592 ± 1.511 mg/100 mg tissue). Capric acid (C10:0) belonging to MC-SFA could be detected only in VAT and ScAT in the HFD-CO group. Other 12 and 14 carbon FA (lauric acid, C12:0; myristic acid, C14:0; myristoleic acid, C14:1) were detected in significantly higher concentrations in VAT and ScAT of the animals in the HFD-CO group compared to the control and other experimental groups. A similar tendency was observed in the content of oleic acid (C18:1n9), which showed the highest content in visceral and subcutaneous adipose tissue from the HFD-OO group (45.749 ± 1.637; 45.750 ± 2.719, respectively) compared to control and other HFD groups. Mice that were fed the HFD-FO diet showed a significantly higher concentration of long-chain *n*-3 PUFA compared to all other groups. EPA (C20:5) could not be detected in VAT and ScAT in any other group, except HFD-FO (VAT: 0.145 ± 0.029; ScAT: 0.106 ± 0.017 mg/100 mg tissue). DHA (C22:6n3) was detected at a 10 times higher concentration in VAT samples in the HFD-FO group than in the control and other HFD groups (0.697 ± 0.126 mg/100 mg tissue), whereas in ScAT, this long-chain polyunsaturated fatty acid could be detected only in the tissue samples isolated from the HFD-FO mice (0.465 ± 0.113 mg/100 mg tissue).

### 3.4. Autophagy Markers in Visceral and Subcutaneous White Adipose Tissue

**Figure 5:**
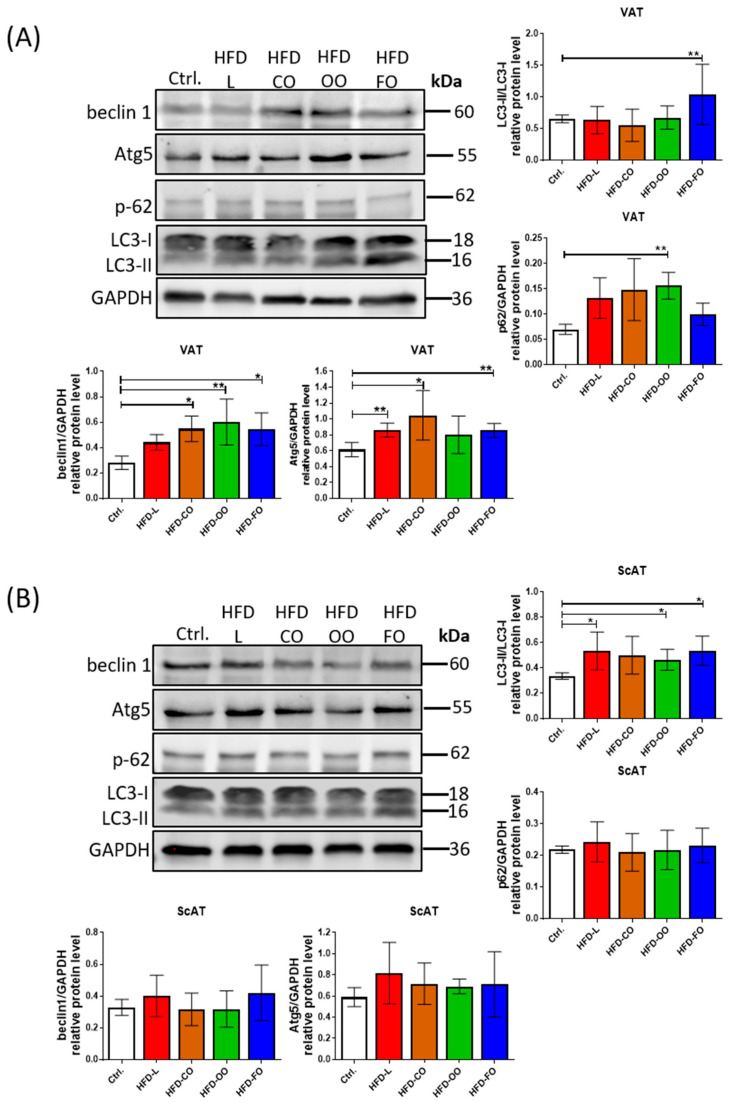
Western blot analysis of the levels of autophagic markers: beclin 1, Atg5, p62 and LC3 in VAT (**A**) and ScAT (**B**) of mice in the control group or different experimental HFD groups. GAPDH was used as the reference protein. Bar graphs show the analysis of integrated optical density (IOD) of each band normalized to IOD of GAPDH, except for LC3, in which case the IOD values represent the ratio: LC3II/LC3I. Densitometry results are presented as means ± SD. Western blot analysis was performed on samples from at least six animals from each group. Symbols * and ** show significant differences (*p* < 0.05 or *p* < 0.01, respectively) between the specified groups.

Hypertrophic adipose tissue in obese individuals is characterized by increased autophagy, although the factors causing autophagy upregulation in the hypertrophic adipocytes are not fully explained. Thus, in the present study, we investigated the effect of different lipid compositions in HFD on autophagy activity in the visceral and subcutaneous adipose tissue of mice taking part in the in vivo experiment. Western blot analysis of chosen autophagic markers in VAT revealed increased levels of beclin 1 and Atg5 in samples from most experimental HFD groups (Figure 5A). These proteins are involved in autophagosome formation. A significant increase in beclin 1 level compared to the control group was noted in VAT samples from the HFD-CO, HFD-OO and HFD-FO groups (HFD-CO vs. Ctrl., *p* = 0.02; HFD-OO vs. Ctrl., *p* = 0.005; HFD-FO vs. Ctrl., *p* = 0.02), whereas in the HFD-L group, the level of this protein was also higher than in the control; however, the result was not significant (HFD-L vs. Ctrl., *p* = 0.25). Atg5 protein also showed a higher level in VAT samples from the HFD groups (HFD-L vs. Ctrl., *p* = 0.002; HFD-CO vs. Ctrl., *p* = 0.03; HFD-FO vs. Ctrl., *p* = 0.003), but the results were insignificant in HFD-OO when compared with the control (HFD-OO vs. Ctrl., *p* = 0.16).

Furthermore, the ratio of LC3-II to LC3-I protein, a key indicator of autophagosome formation, appeared to be altered depending on the lipid composition of the diet. Notably, mice that were fed with the HFD-FO diet exhibited significantly increased LC3-II levels relative to the control and other HFD groups, suggesting enhanced autophagic activity under these experimental conditions (HFD-FO vs. Ctrl., *p* = 0.006) (Figure 5A). In parallel, the level of p62, a substrate degraded during active autophagy, was decreased in the HFD-FO group (showed a similar level to the control group), indicating an increased autophagic flux in VAT of the mice that were fed the high-fat diet containing fish oil. Conversely, the visceral adipose tissue from other HFD groups displayed similar levels of LC3-II/LC3-I ratio with a tendency of increased p62 levels, although the difference was significant compared to control conditions only in the case of the HFD-OO group (HFD-OO vs. Ctrl., *p* = 0.004). These observations indicate suppressed autophagic flux in VAT of mice receiving a high-fat diet containing lard, coconut oil or olive oil as the main dietary source of lipids.

Analysis of autophagic markers in ScAT did not show significant differences among tested groups, despite the observed tendency of increased levels of beclin1 and Atg5 (Figure 5B). The only significant increase was noted with the LC3-II/LC3-I ratio in HFD-L, HFD-OO and HFD-FO when compared to the control (HFD-L vs. Ctrl., *p* = 0.04; HFD-OO vs. Ctrl., *p* = 0.02; HFD-FO vs. Ctrl., *p* = 0.02). However, it was not accompanied with decreased levels of p62 protein in any of the experimental groups, indicating that the activity of autophagic flux was disturbed in the subcutaneous adipose tissue of mice that were fed the high-fat diets, regardless of the lipid origin.

### 3.5. ER Stress Markers in Visceral and Subcutaneous White Adipose Tissue

Autophagy activity is increased under cellular stress conditions, such as ER stress or oxidative stress. Studies have shown that SFA, such as palmitic acid, may induce ER stress in adipocytes, leading to increased autophagy activity. In hypertrophied 3T3-L1 adipocytes, palmitate induces ER stress through the PERK-CHOP pathway, which precedes the activation of autophagy. This autophagic response acts protectively, mitigating PA-induced ER stress and inflammation, highlighting its role in maintaining adipocyte homeostasis [12]. Conversely, omega-3 PUFAs stimulate antioxidant pathways, including AMPK–Nrf2 crosstalk, which reduces reactive oxygen species (ROS) generation and alleviates ER stress, collectively restoring cellular homeostasis and contributing to systemic metabolic improvements [25]. Thus, we also analyzed the levels of chosen ER stress markers in VAT and ScAT of mice in experimental and control groups.

**Figure 6:**
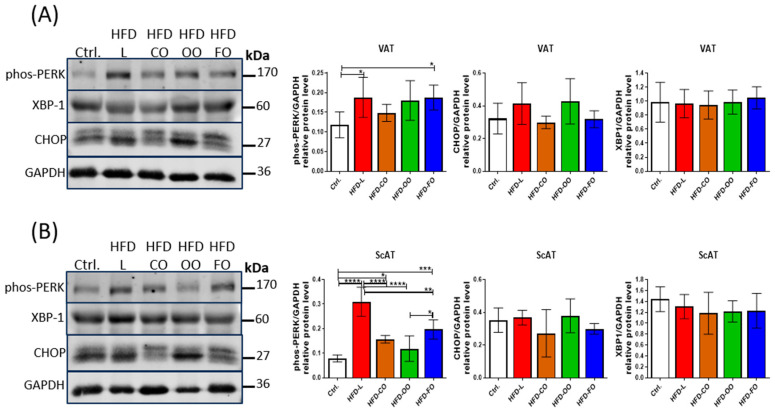
Western blot analysis of the protein levels of ER stress markers: phosphorylated PERK (Thr980), XBP-1 and CHOP in VAT (**A**) and ScAT (**B**) of mice in the control group or different experimental HFD groups. GAPDH was used as the reference protein. Bar graphs show the analysis of integrated optical density (IOD) of each band normalized to IOD of GAPDH. Densitometry results are presented as means ± SD. Western blot analysis was performed on samples from at least six animals from each group. The following symbols show significant differences between the specified groups: *—*p* < 0.05, **—*p* < 0.01, ***—*p* < 0.001, ****—*p* < 0.0001.

No difference was noted in the level of X-box binding protein 1 (XBP1) in either type of analyzed adipose tissue samples. This suggests no effect of the dietary components in the HFDs on activation of the IRE1α signaling pathway, belonging to one of three signaling pathways sensing the unfolded protein response in cells (Figure 6). However, another ER stress sensor, PERK, showed increased phosphorylation/activation in VAT of mice in the HFD-L and HFD-FO groups when compared to the control (HFD-L vs. Ctrl., *p* = 0.03; HFD-FO vs. Ctrl., *p* = 0.03) (Figure 6A). A similar trend of increased levels of phosphorylated PERK, although not significant, was also noted in the HFD-OO group in VAT (HFD-OO vs. Ctrl., *p* = 0.09). In ScAT samples, the highest levels of phosphorylated/active PERK were noted in the HFD-L group, and this result differed significantly from the control group and other experimental HFD groups (HFD-L vs. Ctrl., *p* < 0.0001; HFD-L vs. HD-CO, *p* < 0.0001; HFD-L vs. HFD-OO, *p* < 0.0001; HFD-L vs. HFD-FO, *p* = 0.001) (Figure 6B). Significantly higher levels of phos-PERK compared to the control were also observed in ScAT from HFD-CO (HFD-CO vs. Ctrl., *p* = 0.02) and HFD-FO (HFD-FO vs. Ctrl., *p* = 0.0001) groups; however, these results were significantly lower than in the HFD-L group. The level of phos-PERK in ScAT in the HFD-OO group was similar to the control (HFD-OO vs. Ctrl., *p* = 0.37) and significantly lower than in the subcutaneous adipose tissue samples from HFD-L and HFD-FO groups (HFD-OO vs. HFD-L, *p* < 0.0001; HFD-OO vs. HFD-FO, *p* = 0.015). We also analyzed the level of CHOP, a transcription factor acting downstream of the PERK signaling pathway. CHOP was shown to regulate the expression of autophagic genes, such as *MAP1LC3B* and *ATG5* [26]. CHOP demonstrated a tendency of increased levels in HFD-L and HFD-OO groups in VAT when compared with the control; however, the results were not significant because of high values of standard deviation among analyzed samples (HFD-L vs. Ctrl., *p* = 0.32; HFD-OO vs. Ctrl., *p* =0.95) (Figure 6A). In the case of the HFD-CO and HFD-OO groups, CHOP levels were similar to the control in VAT. In addition, no significant differences in the level of this transcription factor were noted among the tested ScAT samples (Figure 6B).

### 3.6. Oxidative Stress Markers in Visceral and Subcutaneous White Adipose Tissue

**Figure 7:**
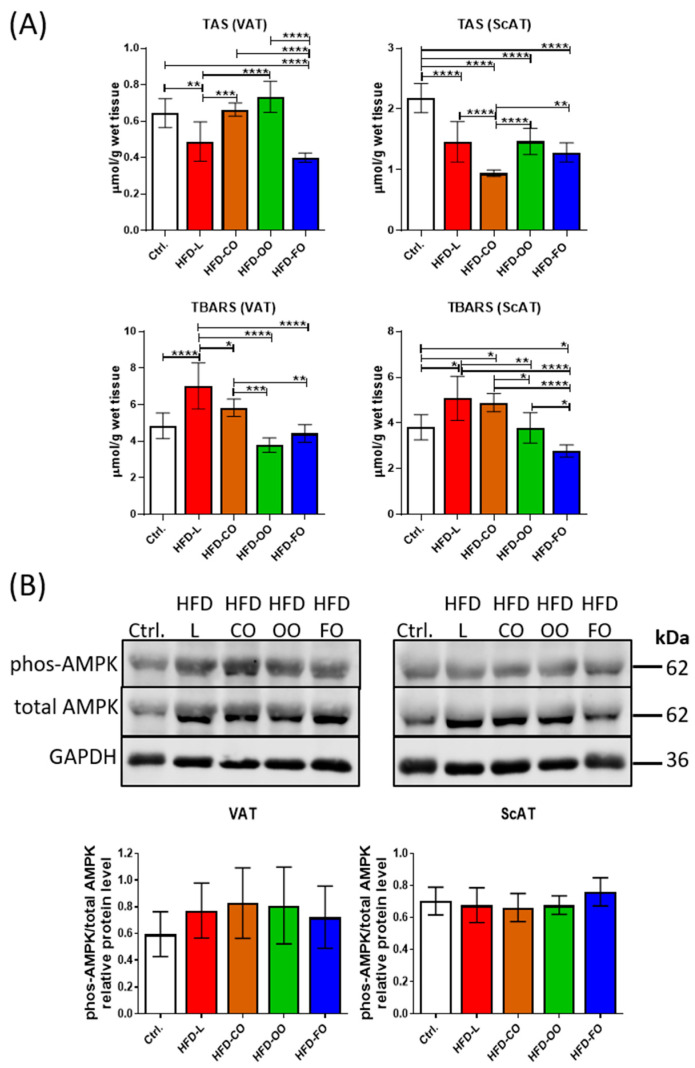
(**A**) Total antioxidative status (TAS) and thiobarbituric acid-reactive substances (TBARSs) used as indicators of lipid peroxidation in VAT and ScAT of mice in the control group or different experimental HFD groups. (**B**) Western blot analysis of phosphorylated AMPK (Thr172) and total AMPK protein in VAT and ScAT of mice in the control group or different experimental HFD groups. GAPDH was used as the reference protein. Bar graphs below the Western blot images show the results presented as IOD ratio of phos-AMPK to total AMPK. TAS, TBARS and Western blot analysis were performed on samples from at least six animals from each group. All results are presented as means ± SD. The following symbols show significant differences between the specified groups: *—*p* < 0.05, **—*p* < 0.01, ***—*p* < 0.001, ****—*p* < 0.0001.

Hypertrophic adipocytes observed in obese subjects show increased oxidative stress, belonging to autophagy inducers in cells. Increased levels of ROS are also among the factors causing WAT dysfunction. We analyzed the total antioxidant status (TAS) and thiobarbituric acid-reactive substances (TBARSs) in both visceral and subcutaneous adipose tissue to assess the oxidative stress (Figure 7). In VAT, TAS was significantly reduced in the HFD-L group compared with control (*p* = 0.008), accompanied by a significant increase in TBARS level (*p* < 0.0001) (Figure 7A). With the HFD-FO group, a significant decrease in TAS was also noted compared to the control (*p* < 0.0001), but TBARS levels remained comparable to the control group (*p* = 0.83). In contrast, both TAS and TBARS in the HFD-CO and HFD-OO groups were similar to those observed in the control. In ScAT, all experimental HFD groups showed significantly (*p* < 0.0001) lower TAS levels than the control group (Figure 7A). The lowest TAS level was noted in the HFD-CO group, and this difference was also significant when compared to other experimental groups (HFD-CO vs. HFD-L, *p* < 0.0001; HFD-CO vs. HFD-OO, *p* < 0.0001; HFD-CO vs. HFD-FO, *p* = 0.005). In parallel, significantly higher values of TBARS were observed in HFD-L and HFD-CO relative to the control (HFD-L vs. Ctrl, *p* = 0.015; HFD-CO vs. Ctrl., *p* = 0.04). On the contrary, TBARS in HFD-FO group showed a significantly lower value than in the control and other HFD groups (HFD-FO vs. Ctrl., *p* = 0.049; HFD-FO vs. HFD-L, *p* < 0.0001; HFD-FO vs. HFD-CO, *p* < 0.0001; HFD-FO vs. OO, *p* = 0.03). The levels of TAS and TBARS in ScAT in HFD-OO group were similar to the values observed in the control group.

In addition to analyses of the total antioxidative status and lipid peroxidation status in VAT and ScAT, we also determined the level of AMPK in both types of adipose tissue by immunoblotting. AMPK is a key energy-sensing kinase that becomes phosphorylated/activated by a reduced level of ATP in cells and serves to restore the correct energy status by several mechanisms, i.a. autophagy activation. AMPK activation can also be stimulated by increased ROS production [27]. In the present study, we did not observe any significant changes in the ratio of phosphorylated to total AMPK levels (phos-AMPK/total AMPK) in both VAT and ScAT samples among analyzed groups (Figure 7B). However, in VAT samples in all experimental HFD groups, a tendency toward increased phos-AMPK/total AMPK ratio was observed when compared with the control group. These results suggest that a high-fat diet, regardless of the lipid composition, did not markedly alter AMPK activation in either adipose tissue depot under the tested conditions.

## 4. Discussion

It is well-established that high-fat diets contribute to the development of obesity [1]. The present study aimed to verify whether the deleterious effects of HFD consumption may be regulated by changing the source of lipids in the diet. We used the DIO model of C57BL/6J mice exposed to high-fat diet in the in vivo experiment. Obesity develops readily in the C57BL/6J strain; significant weight gain occurs within 4 weeks of HFD (45% kcal fat) [28] with increased body fat mass, hyperglycemia, and hyperinsulinemia after 12 weeks [29,30]. The in vivo experiment was divided into two phases. The first phase induced obesity in mice by feeding the animals for 15 weeks with a commonly described HFD with 45% kcal from fat, where lard was the main source of lipids. After 15 weeks, mice in the HFD group already showed significantly increased body mass, significantly elevated glucose concentration in blood, and significantly increased concentration of stearic acid and oleic acid in VAT and ScAT, with an accompanying decrease in linoleic acid content in these tissues (Supplementary Tables S1 and S2), when compared to animals in the control group. Our findings confirmed that the applied HFD model reliably induced the obese phenotype in mice. Subsequently, the second phase of the experiment began, in which mice in the HFD group were divided into four smaller groups (12 animals each) receiving HFD with the same percentage of fat, but differing in the source of lipids. We chose lard (HFD-L), coconut oil (HFD-CO), olive oil (HFD-OO) and fish oil (HFD-FO) as the main sources of fat to test the effect of commonly used dietary lipids. Lard represented fat of animal origin, containing both LC-SFA and MUFA at high concentrations. Coconut oil was chosen because it has been promoted as a functional food in many Western countries, although it contains the highest concentration of SFA among all fat sources, and scientific research has shown that long-term coconut oil consumption leads to increased levels of the low-density lipoprotein cholesterol (LDL) in people [31,32]. Olive oil, one of the main components of the Mediterranean diet, was chosen as a natural source of MUFA at high concentrations, whereas fish oil (represented by cod liver oil in this study) was chosen as the well-documented source of PUFAs, especially the long-chain *n*-3 PUFA.

The HFD-induced obese phenotype in mice was maintained throughout the entire period of the in vivo experiment. Across all experimental groups, body mass was significantly elevated compared with the control group, irrespective of the dietary lipid source. Interestingly, our research showed that mice that were fed the fish oil-based HFD in the second phase of the experiment exhibited a tendency toward reduced weight gain compared with the other HFD groups, although the feed consumption did not vary significantly among animals in different experimental groups. This observation is in line with previous reports demonstrating that long-chain *n*-3 PUFA may exert beneficial effects on lipid metabolism, as well as attenuate systemic inflammation [19], potentially influencing adipose tissue disturbances present in obesity. Our analysis of the fatty acid profile in VAT and ScAT confirmed that the fatty acid composition of adipose tissue closely mirrored the dietary lipid source, albeit with depot-specific variations that may differentially influence adipose tissue function. Particularly noteworthy is the incorporation of long-chain *n*-3 PUFA in the HFD-FO group, where both EPA and DHA were detected in VAT and ScAT, with especially high DHA levels in the visceral adipose tissue.

In obesity, adipose tissue expansion occurs through both hypertrophy, defined as an increase in adipocyte size, and hyperplasia, characterized by an increase in adipocyte number. The relative contribution of these processes to adipose tissue remodeling is modulated by multiple factors, including dietary composition [33]. In our study, the HFD induced pronounced adipose tissue hypertrophy. Whereas no pathological alterations were observed in control animals, focal inflammatory changes were clear in VAT of the HFD-fed mice, supporting the concept that obesity-associated inflammation originates predominantly within visceral depots [4,5]. Adipocyte hypertrophy was evident in both VAT and ScAT across all HFD groups, reflecting a common adaptation to excess lipid storage. Interestingly, the reduction in adipocyte size in ScAT of mice receiving the fish oil-based HFD for the last 12 weeks of the experiment suggests a potential protective effect of long-chain *n*-3 PUFA against adipocyte hypertrophy. This observation is in line with previous reports linking fish oil supplementation to improved adipose tissue function and reduced inflammation [19].

A high-fat diet disrupts glucose homeostasis through combined effects on peripheral insulin sensitivity and pancreatic β-cell function [1]. Elevated circulating free fatty acids, a hallmark of high-fat intake, promote insulin resistance while simultaneously constraining the compensatory capacity of β-cells to maintain adequate insulin secretion. This interplay contributes to the progressive decline in glucose tolerance characteristic of DIO [1]. Previous studies have implicated LC-SFA in this process, likely via mechanisms involving impaired insulin signaling and pro-inflammatory pathways [13]. In contrast, MUFAs derived from olive oil and PUFAs from fish oil have been associated with improved glucose regulation, partly through enhanced insulin sensitivity in obese individuals [6]. Notably, in the second phase of our in vivo experiment, elevated glucose concentration in blood (>150 mg/dL) was maintained only in the HFD-L group, indicating the onset of a prediabetic state in mice that were fed lard-based HFD. While the absence of glucose tolerance testing or insulin measurements prevents definitive conclusions regarding insulin resistance or diabetes development in the present study, our findings are in line with previous reports showing that a diet rich in lard may exacerbate disturbances in glucose metabolism [34,35]. Taken together, our observations demonstrate that the type of dietary lipid exerts a modulatory effect on the body mass and glucose levels when the source of fat is changed after the initial weight gain caused by lard-based HFD, without changing the high lipid content in the diet.

The present study also analyzed the activity of autophagy in VAT and ScAT, as this catabolic process plays a pivotal role in the maintenance of cellular homeostasis. Autophagy is a tightly regulated cellular mechanism responsible for the selective degradation of damaged organelles and cytosolic constituents, including lipid droplets [9]. Chronic overnutrition likely suppresses autophagic and lipophagic activity, promoting lipid accumulation and exacerbating metabolic dysfunction. In obesity, hypertrophic adipocytes experience mitochondrial dysfunction, ROS overproduction, ER stress, and inflammation, all of which promote autophagy. LC3-II protein is the key marker of autophagosome formation that interacts with ER-phagy receptors to facilitate degradation of ER fragments, while beclin 1 regulates initiation of the autophagic cascade [36]. Atg5 is part of ubiquitin-like proteins involved in the next step, elongation of the isolating membrane of the autophagosome. Accumulation of misfolded proteins tagged by p62 further activates autophagy, integrating it with the ubiquitin–proteasome system [36]. Our findings indicate that HFD modulates autophagy in adipose tissue in a lipid source-dependent manner. In VAT, increased levels of beclin 1 and Atg5 across most HFD groups suggest that adipocyte hypertrophy is accompanied by the activation of autophagosome formation, consistent with reports describing autophagy upregulation in obese adipose tissue [10,11]. Notably, the fish oil-based HFD induced a distinct pattern, with a significantly elevated LC3-II/LC3-I ratio and reduced p62 levels, pointing to enhanced autophagic flux. This observation aligns with the proposed protective role of *n*-3 PUFA in promoting cellular homeostasis and mitigating obesity-induced stress in adipose tissue [19,20,21]. Previous studies demonstrated that the presence of EPA and DHA activate AMPK and increase PGC-1α protein level, contributing to increased autophagy activation in cells [22]. Lin et al. [37] showed that EPA promoted autophagy in the nucleus pulposus cells (NPCs), as reflected by increased levels of beclin-1, Atg5, and LC3B-II/LC3-I, along with decreased p62 levels and inhibition of mTORC1 activity. This effect was mediated by AMPK activation, indicating that EPA stimulated autophagy through the AMPK/mTOR signaling axis. Moreover, EPA treatment markedly alleviated induced ER stress in NPCs, as demonstrated by the reduced expression of ER stress markers such as p-PERK/PERK and CHOP [37]. Analysis of ER stress markers in the current study revealed that dietary lipid composition had a differential influence on the unfolded protein response in white adipose tissue. Under ER stress, UPR pathways—PERK, IRE1, and ATF6—modulate autophagy by inducing transcription of autophagy-related genes [38]. While XBP1 levels remained unchanged, suggesting no major activation of the IRE1α pathway, significant alterations were observed in the PERK branch. Specifically, the level of phosphorylated/activated PERK was increased in VAT of mice in all HFD groups, although statistically significant changes were confirmed only in the HFD-L and HFD-FO groups. The downstream transcription factor CHOP showed a trend toward higher levels in VAT in the HFD-L and HFD-OO groups, but variability limited statistical significance. Increased PERK activity corresponded with elevated autophagic flux observed in VAT of mice from the HFD-FO group. Thus, in the presence of PUFA provided with feed, hypertrophic adipocytes may induce functionally active autophagy when the ER-stress is increased. 

Since the AMPK-mediated signaling pathway belongs to the key activators of autophagy in cells [39,40] and AMPK can be activated upon hydrogen peroxide treatment [27], we analyzed the levels of AMPK in the white adipose tissue. Our study did not show changes in the AMPK activation among tested HFD groups and the control group in VAT, suggesting that under the present experimental conditions, HFD did not alter this energy-sensing pathway. However, the present study did not include analyses of mitophagy markers necessary to verify the rate of degradation of damaged mitochondria in cells.

Adipose tissue in obesity is in a chronic inflammatory state that generates high levels of ROS through NF-κB signaling and pro-inflammatory cytokines. Lipid peroxidation products of fatty acids serve as key biomarkers of oxidative stress [3]. Our analysis revealed that the level of oxidative stress parameters (TAS and TBARS) in the white adipose tissue depended not only on the fat content in feed but also on the dietary lipid composition. In VAT, lard-based HFD caused reduced antioxidant capacity and increased lipid peroxidation, supporting the concept that LC-SFA strongly promotes oxidative stress and contributes to visceral adipose tissue dysfunction [41]. The total antioxidative status and the extent of lipid peroxidation were improved when HFD-L was replaced with the olive oil-based HFD. The results obtained emerge from the fact that olive oil contains a high concentration of monounsaturated oleic acid, which is less prone to lipid peroxidation. Furthermore, olive oil is characterized by the presence of biologically active tocopherols and polyphenols that represent minor components (less than 2%) but show strong protective activity against oxidative stress, inhibiting mitochondrial dysfunction, scavenging free radicals, and thus attenuating lipid peroxidation [42,43]. The protective effect against lipid peroxidation was also observed in VAT of mice that were fed the fish oil-based diet in the second phase of the in vivo experiment. Changing the source of lipids in the HFD from lard to fish oil (cod liver oil) rich in *n*-3 PUFA resulted in a significant decrease in TBARS values. These observations are in line with previous reports showing decreased levels of oxidative stress markers in subjects exposed to diets enriched with fish oil [44]. The protective effect of fish oil is attributed mainly to *n*-3 PUFAs, which increase membrane fluidity, improve mitochondrial function and upregulate endogenous antioxidant enzymes such as superoxide dismutase, catalase, and glutathione peroxidase through the activation of transcription factors, such as Nrf2 [45,46]. The expression and activity of enzymes belonging to the endogenous antioxidant system in cells were not analyzed in our study. However, the protective effect of fish oil against oxidative stress observed in the VAT may be linked to enhanced autophagic degradation activity in adipocytes, although AMPK activity was not significantly altered.

The present study did not confirm significant changes in the levels of autophagy and ER stress markers in ScAT. Only PERK activation was significantly increased in the HFD-L group, followed by moderate increases in the HFD-CO and HFD-FO groups, whereas olive oil had little effect. These findings may indicate that LC-SFA, particularly lard, strongly promote ER stress, with potential consequences for adipocyte dysfunction, which is consistent with the currently available literature [12]. The absence of CHOP alterations in ScAT suggests a more resilient response of subcutaneous adipose tissue to dietary lipid-induced ER stress. However, ScAT showed reduced antioxidant capacity manifested by decreased TAS in all HFD groups, and increased TBARS values in HFD-L and HFD-CO groups, both characterized by the high content of SFA. The depot-specific differences observed in our study reinforce the notion that VAT is more susceptible to diet-induced stress, and that the type of dietary fat can modulate the intensity of the cellular response, affecting ER function and antioxidant capacity and modulating the activity of autophagic degradation.

Our study has some limitations that need to be considered. The experiment was performed only on male C57BL/6J mice; therefore, it does not provide information on the sex differences in the body weight variation and distribution, and body composition changes in males and females. Additionally, the glucose tolerance test and insulin tolerance test were not performed over the course of the in vivo experiment; thus, we could not clearly demonstrate that the animals in HFD groups developed insulin resistance or diabetes. Our experiment was planned based on previous reports of other research groups showing that hyperglycemia and impaired glucose tolerance are observed within twelve weeks of feeding HFD with 45 kJ% energy from fat [30]. Future studies should include males and females to delineate whether the changes observed are tissue-specific, or also sex-specific. Another limitation of our research results from the investigation of a chosen, limited number of autophagy-related markers. We included analyses of proteins directly involved in autophagosome formation and elongation (beclin 1, Atg5), and markers of autophagy flux (LC3B-II and p62), but did not show the level of phosphorylated mTOR kinase—the key enzyme inhibiting autophagy induction and an important enzyme regulating cellular metabolism. Further studies should investigate changes in the mTOR activity to reveal in depth the mechanisms of autophagy induction and inhibition. Based on significant differences in oxidative stress among tested HFD groups, future research should also delineate the role of mitophagy in hypertrophic adipocytes, considering that excessive intake of dietary fat leads to chronic inflammation driven by white adipose tissue-derived pro-inflammatory factors.

## 5. Conclusions

The present study confirmed that HFD causes white adipose tissue hypertrophy, regardless of the type of fat present in the diet. Changing the source of dietary lipids after the initial weight gain directly affected the fatty acid composition in the visceral and subcutaneous adipose tissue of animals subjected to the experiment. In addition, antioxidant capacity and autophagy activity in white adipose tissue depended on fat content and lipid source, especially in the visceral depot, whereas autophagy was not significantly affected by the tested high-fat diets in the subcutaneous adipose tissue. Lard-based HFD caused impaired autophagic flux in VAT and increased oxidative stress. Replacing lard with coconut oil or olive oil in the HFD did not improve autophagic degradation activity; however, olive oil increased total antioxidant status and decreased lipid peroxidation in VAT. Replacement of lard with fish oil in the HFD resulted in improved autophagic flux and reduced lipid peroxidation in VAT of mice subjected to dietary intervention. Taken together, the results obtained showed that fish oil, being a good source of long-chain *n*-3 PUFA, induced changes in hypertrophic adipocytes metabolism, especially in the visceral adipose tissue, diminishing the detrimental effects of the high-fat diet initially based mainly on lard. These findings may be relevant for human obesity management, as modulation of dietary lipid composition—particularly increasing long-chain *n*-3 PUFA intake—could improve adipocyte metabolic function, attenuate oxidative stress, and enhance autophagic processes in the visceral fat, a depot closely linked to metabolic syndrome. If confirmed in human adipose tissue, such effects would support the use of *n*-3 PUFA-enriched dietary strategies as adjunctive, non-pharmacological approaches to mitigating obesity-related metabolic disturbances. In addition, the metabolic and autophagic pathways highlighted in this study warrant further investigation in clinical settings, particularly in obese individuals, to determine whether similar depot-specific responses occur in humans.

## Figures and Tables

**Table 1:** Ingredient and caloric composition of the control and experimental high-fat diets used in the in vivo experiment on male C57BL/6j mice. All diets were based on the AIN-93G formulation and contained equivalent caloric content (4057 kcal/kg). Experimental groups differed in the dominant lipid source: lard (HFD-L), coconut oil (HFD-CO), olive oil (HFD-OO), or cod liver oil (HFD-FO), while the control diet (Ctrl.) included only soybean oil and minimal lard. Macronutrient sources, micronutrient mixes, and fiber content were held constant across all diets.

Diet Type	Ctrl.		HFD-L		HFD-CO		HFD-OO		HFD-FO	
Feed Composition	g	kcal	g	kcal	g	kcal	g	kcal	g	kcal
Corn starch	506.2	2024.8	137.2	548.8	137.2	548.8	137.2	548.8	137.2	548.8
Casein (>85% protein)	200.0	800.0	200.0	800.0	200.0	800.0	200.0	800.0	200.0	800.0
Maltodextrin	125.0	500.0	125.0	500.0	125.0	500.0	125.0	500.0	125.0	500.0
Saccharose	68.8	275.2	68.8	275.2	68.8	275.2	68.8	275.2	68.8	275.2
Soybean oil	25.0	225.0	25.0	225.0	25.0	225.0	25.0	225.0	25.0	225.0
Lard	20.0	180.0	184.0	1656.0	59.0	531.0	59.0	531.0	59.0	531.0
Coconut oil	0.0	0.0	0.0	0.0	125.0	1125.0	0.0	0.0	0.0	0.0
Olive oil	0.0	0.0	0.0	0.0	0.0	0.0	125.0	1125.0	0.0	0.0
Cod liver oil	0.0	0.0	0.0	0.0	0.0	0.0	0.0	0.0	125.0	1125.0
Fiber (α-cellulose)	50.0	0.0	50.0	0.0	50.0	0.0	50.0	0.0	50.0	0.0
AIN-93G-Mineral Mix	35.0	0.0	35.0	0.0	35.0	0.0	35.0	0.0	35.0	0.0
AIN-93G-Vitamin Mix	10.0	40.0	10.0	40.0	10.0	40.0	10.0	40.0	10.0	40.0
L-cystine	3.0	12.0	3.0	12.0	3.0	12.0	3.0	12.0	3.0	12.0
Choline Bitartrate	2.5	0.0	2.5	0.0	2.5	0.0	2.5	0.0	2.5	0.0
Tert-butylohydrochinon	0.014	0.0	0.014	0.0	0.014	0.0	0.014	0.0	0.014	0.0
**Total**	1045.5	4057.0	840.5	4057.0	840.5	4057.0	840.5	4057.0	840.5	4057.0

**Table 2:** Fatty acids (FAs) composition in control (Ctrl.) and experimental feeds used in the second stage of in vivo experiment on mice. Symbols of diets: HFD-L (lard as the dominant component); HFD-CO (coconut oil as the dominant component); HFD-OO (olive oil as the dominant component); HFD-FO (fish oil as the dominant component). FA concentration is expressed as mg/g of feed. Meaning of abbreviation: n.d.—not detected (below the detection limit).

		FA Content (mg/1 g Feed)				
FA Omega Nomenclature	Common Name	Ctrl.	HFD-L	HFD-CO	HFD-OO	HFD-FO
C6:0	Caproic acid	n.d.	n.d.	0.90	n.d.	n.d.
C8:0	Caprylic acid	n.d.	n.d.	14.26	n.d.	n.d.
C10:0	Capric acid	0.31	n.d.	12.77	0.24	n.d.
C12:0	Lauric acid	0.38	0.29	105.78	1.46	n.d.
C14:0	Myristic acid	5.16	0.76	48.99	2.23	12.48
C14:1	Myristoleic acid	n.d.	n.d.	n.d.	n.d.	0.68
C15:0	Pentadeclic acid	0.21	n.d.	n.d.	n.d.	0.84
C16:0	Palmitic acid	46.46	90.13	29.67	31.25	32.44
C16:1n10	Sapienic acid	n.d.	n.d.	n.d.	n.d.	1.04
C16:1n9	Elaidic acid	1.39	0.21	0.42	0.58	1.96
C16:1n7	Palmitoleic acid	9.17	0.89	2.78	4.54	24.19
C17:0	Margaric acid	1.33	0.28	0.52	0.69	2.12
C17:1	Margaroleic acid	1.15	0.24	0.36	0.59	2.61
C18:0	Stearic acid	59.03	67.39	37.76	33.45	35.34
C18:1T	Vaccenic acid	0.54	n.d.	n.d.	n.d.	n.d.
C18:1n9	Oleic acid	159.66	188.48	69.51	212.56	109.73
C18:1n3	15E-octadecenoic acid	14.54	2.45	5.51	12.06	23.06
C18:2n7	Rumenic acid	n.d.	n.d.	n.d.	n.d.	0.81
C18:2n6	Linoleic acic	83.43	49.27	48.84	56.58	53.20
C18:3n3 (ALA)	α-Linolenic acid	9.18	4.87	5.69	6.71	8.49
C18:3n6 (GLA)	γ-Linolenic acid	n.d.	n.d.	n.d.	n.d.	6.23
C20:0	Arachidic acid	0.48	0.21	0.33	0.85	0.52
C20:1n7	Paullinic acid	4.57	0.61	1.28	1.79	48.32
C20:2n6	*Cis*-11,14-Eicosadienoic Acid	2.74	0.33	0.64	0.55	1.58
C20:3n9	Mead acid	n.d.	0.41	n.d.	0.71	n.d.
C20:4n6	Arachidonic Acid	1.97	n.d.	n.d.	1.98	40.16
C20:5n3	Eicosapentaenoic acid	n.d.	n.d.	n.d.	n.d.	21.16
C21:0	Heneicosanoic acid	n.d.	n.d.	n.d.	n.d.	2.04
C22:5n3	Docosapentaenoic Acid	n.d.	n.d.	n.d.	n.d.	7.31
C22:6n3	Docosahexaenoic acid	n.d.	n.d.	n.d.	n.d.	48.67
C24:1n9	Nervonic acid	n.d.	n.d.	n.d.	n.d.	3.44

**Table 3:** List of antibodies used in immunoblotting analyses.

Name	Producer	Catalog Number	Dilution
Atg5 rabbit mAb	Cell Signaling Technology	sc-12994	1:500
Beclin1 rabbit mAb	Cell Signaling Technology	sc-3495	1:500
LC3B rabbit polyclonal Ab	Cell Signaling Technology	sc-2775	1:500
Phoso PERK (Thr980) rabbit mAB	Cell Signaling Technology	sc-3179	1:500
XBP-1s rabbit mAb	Cell Signaling Technology	sc-40435	1:500
CHOP mouse mAb	Cell Signaling Technology	sc-2895	1:500
Anti-phoso AMPKα (Thr172) rabbit mAB	Cell Signaling Technology	sc-2535	1:500
AMPK rabbit polyclonal Ab	Cell Signaling Technology	sc-2532	1:500
GAPDH Loading Control mAb	Invitrogen, Thermo Fisher Scientific	MA515738	1:2000
Secondary IRDye^®^ 800CW donkey anti-Rabbit IgG (H + L)	LI-COR	926-32213	1:5000
Secondary IRDye^®^ 680LT Donkey anti-Mouse IgG (H + L)	LI-COR	926-68022	1:5000

**Table 4:** Content of fatty acids (FAs) in visceral white adipose tissue (VAT) of mice fed a control diet (Ctrl.) or high-fat diet (HFD) with a different composition of lipids: HFD-L (lard as dominant component); HFD-CO (coconut oil as dominant component); HFD-OO (olive oil as dominant component); HFD-FO (fish oil as dominant component). Values are expressed as mg FA per 100 mg of tissue (mean ± SD). Symbols: * and ^#^ in superscripts represent statistically significant differences (*p* ≤ 0.05) from Ctrl. or HFD-L, respectively. Meaning of abbreviation: n.d.—not detected (below the detection limit).

		FA Content in VAT (mg/100 mg Tissue)				
FA Omega Nomenclature	Common Name	Ctrl.	HFD-L	HFD-CO	HFD-OO	HFD-FO
C10:0	Capric acid	n.d.	n.d.	0.168 ± 0.020	n.d.	n.d.
C12:0	Lauric acid	0.039 ± 0.005	0.033 ± 0.009	4.040 *^#^ ± 0.401	0.040 ± 0.018	0.033 ± 0.005
C14:0	Myristic acid	0.756 ^#^ ± 0.101	0.482 * ± 0.083	2.965 *^#^ ± 0.250	0.348 * ± 0.035	0.962 *^#^ ± 0.196
C14:1	Myristoleic acid	0.056 ± 0.013	n.d.	0.153 ± 0.032	n.d.	n.d.
C15:0	Pentadeclic acid	0.080 ^#^ ± 0.010	0.041 * ± 0.010	0.054 *^#^ ± 0.007	0.036 * ± 0.008	0.094 *^#^ ± 0.013
C16:0	Palmitic acid	7.521 ^#^ ± 0.165	6.411 * ± 0.344	5.758 *^#^ ± 0.707	5.646 *^#^ ± 0.488	6.100 * ± 0.386
C16:1n10	Sapienic acid	n.d.	n.d.	n.d.	n.d.	0.253 ± 0.058
C16:1n9	Elaidic acid	0.741 ^#^ ± 0.061	1.038 * ± 0.075	0.866 *^#^ ± 0.158	1.143 * ± 0.134	1.150 * ± 0.197
C16:1n7	Palmitoleic acid	7.265 ^#^ ± 1.123	3.141 * ± 0.279	5.117 *^#^ ± 0.583	3.024 * ± 0.561	3.727 * ± 0.618
C17:0	Margaric acid	0.088 ^#^ ± 0.017	0.116 * ± 0.015	0.065 *^#^ ± 0.009	0.080 ^#^ ± 0.006	0.181 *^#^ ± 0.035
C17:1	Margaroleic acid	0.207 ^#^ ± 0.021	0.163 * ± 0.016	0.144 * ± 0.013	0.118 *^#^ ± 0.017	0.214^#^ ± 0.039
C18:0	Stearic acid	1.490 ^#^ ± 0.112	2.438 * ± 0.235	1.349 ^#^ ± 0.224	1.751 *^#^ ± 0.097	2.495 * ± 0.348
C18:1T	Vaccenic acid	n.d.	0.056 ± 0.004	n.d.	n.d.	n.d.
C18:1n9	Oleic acid	31.287 ^#^ ± 3.407	37.652 * ± 1.559	31.520 ^#^ ± 2.787	45.749 *^#^ ± 1.637	33.489 ^#^ ± 2.861
C18:1n3	15E-octadecenoic acid	7.554 ^#^ ± 0.666	5.597 * ± 0.555	5.025 * ± 1.178	4.930 * ± 0.560	6.306 *^#^ ± 0.456
C18:2n6	Linoleic acic	14.666 ^#^ ± 1.627	12.146 * ± 0.507	9.901 *^#^ ± 1.259	10.432 *^#^ ± 0.960	10.385 *^#^ ± 0.456
C18:3n3 (ALA)	α-Linolenic acid	0.687 ^#^ ± 0.083	0.338 * ± 0.082	0.328 * ± 0.030	0.292 * ± 0.034	0.480 *^#^ ± 0.124
C18:3n6 (GLA)	γ-Linolenic acid	n.d.	n.d.	n.d.	n.d.	0.085 ± 0.044
C20:0	Arachidic acid	0.039 ^#^ ± 0.011	0.018 * ± 0.005	0.020 * ± 0.005	0.017 * ± 0.003	0.019 * ± 0.005
C20:1n7	Paullinic acid	0.263 ^#^ ± 0.040	0.498 * ± 0.079	0.456 ± 0.086	0.387 ± 0.067	3.414 *^#^ ± 0.424
C20:2n6	*Cis*-11,14-Eicosadienoic Acid	0.066 ^#^ ± 0.022	0.116 * ± 0.007	0.057 ^#^ ± 0.010	0.051 ^#^ ± 0.020	0.106 * ± 0.023
C20:3n9	Mead acid	0.186 ^#^ ± 0.022	0.028 * ± 0.008	0.030 * ± 0.008	0.023 * ± 0.006	0.514 *^#^ ± 0.023
C20:4n6	Arachidonic Acid	0.134 ± 0.024	0.105 ± 0.042	0.094 * ± 0.029	0.073 *^#^ ± 0.018	0.079 *^#^ ± 0.029
C20:5n3	Eicosapentaenoic acid	n.d.	n.d.	n.d.	n.d.	0.145 ± 0.029
C22:5n3	Docosapentaenoic Acid	n.d.	n.d.	n.d.	n.d.	0.248 ± 0.041
C22:6n3	Docosahexaenoic acid	0.070 ± 0.010	0.021 ± 0.008	0.067 * ± 0.035	n.d.	0.697 *^#^ ± 0.126

**Table 5:** Content of fatty acids (FAs) in subcutaneous white adipose tissue (ScAT) of mice fed a control diet (Ctrl.) or high-fat diet (HFD) with a different composition of lipids: HFD-L (lard as dominant component); HFD-CO (coconut oil as dominant component); HFD-OO (olive oil as dominant component); HFD-FO (fish oil as dominant component). Values are expressed as mg FA per 100 mg of tissue (mean ± SD). Symbols: * and ^#^ in superscripts represent statistically significant differences (*p* ≤ 0.05) from Ctrl. or HFD-L, respectively. Meaning of abbreviation: n.d.—not detected (below the detection limit).

		FA Content in ScAT (mg/100 mg Tissue)				
FA Omega Nomenclature	Common Name	Ctrl.	HFD-L	HFD-CO	HFD-OO	HFD-FO
C10:0	Capric acid	n.d.	n.d.	0.140 ± 0.021	n.d.	n.d.
C12:0	Lauric acid	0.038 ± 0.010	0.031 ± 0.009	3.998 *^#^ ± 0.472	0.036 ± 0.012	0.020 ± 0.009
C14:0	Myristic acid	0.744 ^#^ ± 0.080	0.482 * ± 0.122	3.092 *^#^ ± 0.290	0.312 *^#^ ± 0.035	0.740 ^#^ ± 0.159
C14:1	Myristoleic acid	0.063 ^#^ ± 0.009	0.014 * ± 0.009	0.171 *^#^ ± 0.026	n.d.	0.012 *^#^ ± 0.005
C15:0	Pentadeclic acid	0.066 ^#^ ± 0.005	0.044 * ± 0.012	0.054 *^#^ ± 0.008	0.032 *^#^ ± 0.007	0.081 *^#^ ± 0.015
C16:0	Palmitic acid	5.540 ^#^ ± 0.471	7.135 * ± 1.134	6.085 ^#^ ± 0.337	5.496 ^#^ ± 0.457	5.493 ^#^ ± 1.407
C16:1n10	Sapienic acid	n.d.	n.d.	n.d.	n.d.	0.232 ± 0.052
C16:1n9	Elaidic acid	0.718 ^#^ ± 0.058	1.014 * ± 0.187	0.900 * ± 0.129	1.034 * ± 0.058	0.897 * ± 0.238
C16:1n7	Palmitoleic acid	6.186 ^#^ ± 0.968	3.319 * ± 0.863	5.991 ^#^ ± 0.276	2.406 *^#^ ± 0.377	3.018 * ± 0.800
C17:0	Margaric acid	0.066 ^#^ ± 0.011	0.131 * ± 0.023	0.077 ^#^ ± 0.014	0.084 ^#^ ± 0.014	0.181 *^#^ ± 0.041
C17:1	Margaroleic acid	0.157 ^#^ ± 0.015	0.185 * ± 0.035	0.172 ± 0.021	0.104 *^#^ ± 0.011	0.153 ^#^ ± 0.023
C18:0	Stearic acid	1.098 ^#^ ± 0.246	3.010 * ± 0.500	1.580 *^#^ ± 0.185	2.018 *^#^ ± 0.280	2.563 *^#^ ± 0.686
C18:1n9	Oleic acid	26.555 ^#^ ± 1.910	39.562 * ± 4.609	32.947 *^#^ ± 2.772	45.750 *^#^ ± 2.719	32.637 *^#^ ± 3.658
C18:1n3	15E-octadecenoic acid	6.321 ± 1.036	6.211 ± 0.831	5.729 ± 0.776	5.284 *^#^ ± 0.968	6.571 ± 0.500
C18:2n6	Linoleic acic	11.719 ^#^ ± 0.845	13.592 * ± 1.511	10.811 *^#^ ± 0.745	9.744 *^#^ ± 0.592	9.301 *^#^ ± 1.293
C18:3n3 (ALA)	α-Linolenic acid	0.456 ^#^ ± 0.053	0.380 * ± 0.123	0.375 * ± 0.032	0.259 *^#^ ± 0.044	0.361 * ± 0.092
C18:3n6 (GLA)	γ-Linolenic acid	n.d.	n.d.	n.d.	n.d.	0.048 ± 0.014
C20:0	Arachidic acid	0.027 ^#^ ± 0.008	0.017 * ± 0.002	0.019 ± 0.005	0.013 * ± 0.005	0.013 * ± 0.006
C20:1n7	Paullinic acid	0.502 ± 0.109	0.571 ± 0.140	0.438 ± 0.216	0.402 ± 0.087	3.351 *^#^ ± 0.773
C20:2n6	*Cis*-11,14-Eicosadienoic Acid	n.d.	0.111 ± 0.042	0.055 ^#^ ± 0.017	0.037 ^#^ ± 0.004	0.071 ^#^ ± 0.019
C20:3n9	Mead acid	0.026 ± 0.008	0.030 ± 0.015	0.024 ± 0.007	0.015 ± 0.005	0.401 *^#^ ± 0.127
C20:5n3	Eicosapentaenoic acid	n.d.	n.d.	n.d.	n.d.	0.106 ± 0.017
C22:1n9	Erucic acid	0.097 ± 0.025	0.085 ± 0.034	0.081 ± 0.016	0.043 * ± 0.009	0.398 *^#^ ± 0.133
C22:6n3	Docosahexaenoic acid	n.d.	n.d.	n.d.	n.d.	0.465 ± 0.113

## Data Availability

The data presented in this study are available in the article. Further inquiries can be directed to the corresponding author. The data presented constitute part of the results obtained in the research currently being conducted.

## Supplementary Materials

The following supporting information can be downloaded at https://www.mdpi.com/article/10.3390/nu17233776/s1, Table S1: Content of fatty acids (FAs) in the visceral white adipose tissue (VAT) of mice that were fed a control diet (Ctrl.) or high-fat diet with lard as the dominant component for 15 weeks (HFD-L). The table summarizes the first phase of dietary intervention. Results are presented as means ± standard deviation. Bold font is used to show the most abundant fatty acids detected in VAT. Symbol: * in a superscript represents a statistically significant difference from Ctrl.: ** *p* < 0.01, *** *p* < 0.001, **** *p* < 0.0001. Table S2: Content of fatty acids (FAs) in the subcutaneous white adipose tissue (ScAT) of mice fed a control diet (Ctrl.) or high-fat diet with lard as the dominant component for 15 weeks (HFD-L). The table summarizes the first phase of dietary intervention. Results are presented as means ± standard deviation. Bold font is used to show the most abundant fatty acids detected in ScAT. Symbol: * in a superscript represents a statistically significant difference from Ctrl.: * *p* < 0.05, ** *p* < 0.01, **** *p* < 0.0001.

## Abbreviations

The following abbreviations are used in this manuscript:

DIO	Diet-Induced Obesity
HFD	High-Fat Diet
LC-SFA	Long-chain Saturated Fatty Acids
MC-SFA	Medium-Chain Saturated Fatty Acids
MUFA	Monounsaturated Fatty acids
PUFA	Polyunsaturated Fatty Acid
ER	Endoplasmic Reticulum
UPR	Unfolded Protein Response
ROS	Reactive Oxygen Species
AMPK	AMP-dependent Protein Kinase
PERK	Protein Kinase RNA-like ER kinase
CHOP	C/EBP homologous protein
XBP1	X-box binding protein 1
