# Supplementary material for: Fish Oil Present in High-Fat Diet, Unlike Other Fats, Attenuates Oxidative Stress and Activates Autophagy in Murine Adipose Tissue
Source: Nutrients. 2025 Dec 1;17(23):3776. doi: 10.3390/nu17233776 (PMC12693979; doi:10.3390/nu17233776)
Supplement: Supplementary file 1 [file nutrients-17-03776-s001.zip › Table S1_FA concentration in VAT_15w_nutrients.pdf]

Table S1. Content of fatty acids (FA) in the visceral white adipose tissue (VAT) of mice fed for 15 weeks a control diet (Ctrl.) or high fat diet with lard as dominant component (HFD-L). The table summarizes the first phase of dietary intervention. Results are presented as means ± standard deviation. Bold font is used to show the most abundant fatty acids detected in VAT. Symbol: * in a superscript represents a statistically significant difference from Ctrl.: $^{**}p < 0.01$, $^{***}p < 0.001$, $^{****}p < 0.0001$.

FA content in VAT (mg/100 mg tissue)			
**FA Omega Nomenclature**	**Common name**	**Ctrl.**	**HFD-L**
C12:0	Lauric acid	0.044 ± 0.005	0.028 ± 0.003
C14:0	Myristic acid	0.747 ± 0.052	0.610 ± 0.028
C14:1	Myristoleic acid	0.058 ± 0.018	0.019 ± 0.003
C15:0	Pentadeclic acid	0.088 ± 0.008	0.050 ± 0.005
**C16:0**	**Palmitic acid**	**6.457 ± 0.343**	**7.258 ± 0.338****
C16:1n9	Elaidic acid	0.774 ± 0.031	0.657 ± 0.050
**C16:1n7**	**Palmitoleic acid**	**5.853 ± 0.272**	**4.090 ± 0.272******
C17:0	Margaric acid	0.100 ± 0.005	0.123 ± 0.010
C17:1	Margaroleic acid	0.194 ± 0.013	0.185 ± 0.008
**C18:0**	**Stearic acid**	**1.539 ± 0.096**	**2.605 ± 0.233******
**C18:1n9**	**Oleic acid**	**28.603 ± 1.202**	**32.560 ± 2.113****
**C18:1n3**	**15E-octadecenoic acid**	**5.925 ± 0.425**	**4.362 ± 0.536*****
**C18:2n6**	**Linoleic acic**	**16.045 ± 0.954**	**12.564 ± 0.537******
C18:3n3 (ALA)	α-Linolenic acid	0.687$^{\#}$ ± 0.083	0.338* ± 0.082
C18:3n6 (GLA)	γ-Linolenic acid	0.722 ± 0.080	0.547 ± 0.045
C20:0	Arachidic acid	0.052 ± 0.006	0.027 ± 0.002
C20:1n7	Paullinic acid	0.781 ± 0.023	0.530 ± 0.070
C20:2n6	*Cis*-11,14-Eicosadienoic Acid	0.083 ± 0.007	0.150 ± 0.009
C20:3n9	Mead acid	0.189 ± 0.021	0.145 ± 0.031
C20:4n6	Arachidonic Acid	0.134 ± 0.024	0.105 ± 0.042
C22:6n3	Docosahexaenoic acid	0.041 ± 0.012	0.025 ± 0.014
