# Supplementary material for: Fish Oil Present in High-Fat Diet, Unlike Other Fats, Attenuates Oxidative Stress and Activates Autophagy in Murine Adipose Tissue
Source: Nutrients. 2025 Dec 1;17(23):3776. doi: 10.3390/nu17233776 (PMC12693979; doi:10.3390/nu17233776)
Supplement: Supplementary file 1 [file nutrients-17-03776-s001.zip › Table S2_FA concentration in ScAT_15w_nutrients.pdf]

Table S2. Content of fatty acids (FA) in the subcutaneous white adipose tissue (ScAT) of mice fed for 15 weeks a control diet (Ctrl.) or high fat diet with lard as dominant component (HFD-L). The table summarizes the first phase of dietary intervention. Results are presented as means ± standard deviation. Bold font is used to show the most abundant fatty acids detected in ScAT. Symbol: * in a superscript represents a statistically significant difference from Ctrl.: *p < 0.05, ** p < 0.01, **** p < 0.0001.

FA content in VAT (mg/100 mg tissue)			
**FA Omega Nomenclature**	**Common name**	**Ctrl.**	**HFD-L**
C12:0	Lauric acid	0.044 ± 0.012	0.079 ± 0.069
C14:0	Myristic acid	0.768 ± 0.175	0.564 ± 0.156
C14:1	Myristoleic acid	0.044 ± 0.009	0.154 ± 0.020
C15:0	Pentadeclic acid	0.056 ± 0.019	0.049 ± 0.010
**C16:0**	**Palmitic acid**	**5.578 ± 1.342**	**7.257 ± 1.424**
C16:1n9	Elaidic acid	0.530 ± 0.109	0.687 ± 0.082
**C16:1n7**	**Palmitoleic acid**	**4.998 ± 1.022**	**3.541 ± 0.932** *
C17:0	Margaric acid	0.059 ± 0.020	0.130 ± 0.029
C17:1	Margaroleic acid	0.088 ± 0.032	0.226 ± 0.036
**C18:0**	**Stearic acid**	**1.275 ± 0.422**	**3.265 ± 0.642** ****
**C18:1n9**	**Oleic acid**	**23.071 ± 5.809**	**33.494 ± 5.067** **
**C18:1n3**	**15E-octadecenoic acid**	**5.513 ± 1.708**	**5.920 ± 0.710**
**C18:2n6**	**Linoleic acic**	**11.474 ± 3.636**	**11.950 ± 2.395**
C18:3n3 (ALA)	α-Linolenic acid	0.390 ± 0.058	0.490 ± 0.186
C20:0	Arachidic acid	1.088 ± 0.595	0.021 ± 0.011
C20:1n7	Paullinic acid	0.553 ± 0.270	0.457 ± 0.189
C20:2n6	*Cis*-11,14-Eicosadienoic Acid	0.018 ± 0.014	0.120 ± 0.046
C20:3n9	Mead acid	0.014 ± 0.003	0.031 ± 0.002
C22:1n9	Erucic acid	0.038 ± 0.026	0.119 ± 0.024
